# Immunoregulation of NKT Cells in Systemic Lupus Erythematosus

**DOI:** 10.1155/2015/206731

**Published:** 2015-12-24

**Authors:** Junwei Chen, Meng Wu, Jing Wang, Xiaofeng Li

**Affiliations:** Department of Rheumatology, The Second Hospital of Shanxi Medical University, Shanxi, Taiyuan 030001, China

## Abstract

Systemic lupus erythematosus (SLE) is a multisystem autoimmune disease with different variety of clinical manifestations. Natural killer T (NKT) cells are innate lymphocytes that play a regulatory role during broad range of immune responses. A number of studies demonstrated that the quantity and quality of invariant NKT (iNKT) cells showed marked defects in SLE patients in comparison to healthy controls. This finding suggests that iNKT cells may play a regulatory role in the occurrence and development of this disease. In this review, we mainly summarized the most recent findings about the behavior of NKT cells in SLE patients and mouse models, as well as how NKT cells affect the proportion of T helper cells and the production of autoreactive antibodies in the progress of SLE. This will help people better understand the role of NKT cells in the development of SLE and improve the therapy strategy.

## 1. Introduction

Systemic lupus erythematosus (SLE) is a chronic autoimmune inflammatory disease that is characterized by involvement in multiple organs and the overproduction of autoantibodies. The serological symbol of SLE is the redundant production of autoantibodies against the antigens that locate within the nucleus of cells, such as double-stranded DNA (dsDNA), which is the dominant antigen of SLE. These autoantibodies are deposited within the capillaries of multiple organs with self-antigens and subsequently result in systemic disorders.

Although the precise pathogenesis of SLE remains unclear, abnormal immune tolerance and overactivation or hyperproliferation of T and B lymphocytes are considered to be some of the main causes. That B cells can produce multiple autoantibodies against autoantigens with help from T cells that have been verified by a number of studies in vivo or in vitro [1]. To avoid the development of autoimmune disease, the tolerance to autoantigens should be established either before or after the initiation of an autoreactive response [2]. An important mechanism of tolerance is anergy, which is the inability to react with some specific antigens (i.e., a state of unresponsiveness to a specific antigen under certain conditions) [3]. Another primary mechanism of tolerance is the suppression of excessive immune response among conventional T cells, such as regulatory CD4+CD25+ T cells (Tregs) [4].

In addition to B cells and T cells, related cells of the innate immune system, such as NKT cells, can sense microbial pathogens by responding to conserved pathogen-associated molecular patterns. Recent studies demonstrated that NKT cells play a suppressive role in chronic inflammatory diseases, such as SLE [5], rheumatoid arthritis (RA) [6], Sjögren's syndrome (SS) [7], systemic sclerosis (SSc) [8], psoriasis (PSA) [9], adult onset Still's disease (AOSD) [10], and Behcet's uveitis [11]. NKT lymphocytes are one subset of T lymphocytes that possess features of both natural killer (NK) cells and conventional T lymphocytes [12, 13]. The generation, differentiation, and progress of NKT lymphocytes occur in the thymus by positive selection, negative selection, and VDJ recombination [14]. Most NKT cells express an invariant T cell receptor (TCR) that is integrated by the combination of V*α*14-J*α*18 and V*β*8.2/V*β*7/V*β*2 chains in mice and homologous V*α*24-J*α*18 and V*β*11 chains in humans, unlike conventional adaptive cells, which express diverse TCRs [15, 16]. NKT cells are mainly divided into three subtypes according to the molecular markers. The first group is the conserved major histocompatibility complex (MHC) class I-like molecule CD1d-dependent which is called iNKT cells or type I NKT cells, because there is invariant T cell receptor *α* chain (TCR-*α*) on the surface of the cells to recognize the CD1d molecules unlike conventional T cells. Another group is the CD1d-independent NKT-like cells called type II NKT cells. Although these two kinds of NKT cells both express TCR-*α*, the TCR repertoire is various, not invariant. And the diverse TCRs are not capable of recognizing the CD1d. The TCRs can recognize other antigens which may be not antigen specific [17, 18]. Recent studies have reported that some iNKT cells can secrete IL-17 like Th17 cells. It has been identified as NKT17 cells due to production of IL-17, the third subtype NKT cells [19]. Type I NKT cells, that is, iNKT cells, possess the features of innate immune cells and adaptive immune cells. iNKT cells are easy to be activated by endogenous or exogenous glycolipids via their TCRs. These energetic cells consequently induce immune lymphocytes activation including autologous T, B lymphocytes. Therefore iNKT cells play an important role in immune response [20, 21]. It has been assumed that iNKT cells can bridge the innate and adaptive immune response; moreover there are some evidences confirming that iNKT cells are involved in the inflammatory response [22]. Even though type II NKT cells and NKT17 cells are implicated, we reasonably think that iNKT cells play an important role in immune response including autoimmune diseases. iNKT cells come from thymocytes in thymus. These cells undergo positive selection through CD4+CD8+ thymocytes. Some evidences confirmed that iNKT cells migrate from cortex to medulla. During thymocyte development, the progenitors express high level of CD4 and lesser level of CD161, whereas differentiated iNKT cells exhibit a downregulation of CD4 expression and an upregulation of CD161 expression. The differentiation of iNKT cells is companied by the changes of molecular markers on the cells in the process of maturation. Many studies suggested the differentiation of iNKT cells depended on the surroundings in thymus. The different conditions result in different expression on these cells, which determine the specific subtype of NKT cells [23]. iNKT cells can generate diverse cytokines depending on the different environment [24]. Indeed, iNKT cells rapidly exert effector functions during an immune response without the requirement for further activation. If iNKT cells are further stimulated by glycolipid ligands, they can produce a burst of cytokines and chemokines that endow these cells with potent immunomodulatory activities. iNKT cells are able to secrete considerable immunoregulatory cytokines, such as IL-2, IL-4, IL-6, IL-10, IL-13, IL-17, IL-21, IFN-*γ*, TGF-*β*, TNF-*α*, and GM-CSF, which are implicated in a variety of different immune reactions [25]. That NKT cells can interact with other immune cells, such as T cells, B cells, NK cells, and dendritic cells (DCs), has been confirmed [26]. Although the interaction between NKT cells and other immune cells is mediated by cytokines, some studies indicated that they can be contacted by cell-to-cell [13, 27, 28]. Therefore, NKT cells can play a role by interaction with other immune cells in many immune responses, including inflammatory diseases [29]. Although the regulatory effects of iNKT cells have already been expounded in some diseases, these regulatory effects of these cells in SLE have not been well summarized. This review focuses on the relationship between the iNKT cell population and the development of SLE, on how iNKT cells are involved in the regulation of SLE by shaping immune responses, and on potential applications of these cells in SLE treatment.

## 2. The Correlation of the iNKT Population and the Development of Systemic Lupus Erythematosus

The number and proportion of iNKT cells were shown to significantly decrease in both patients with SLE and the mouse lupus models [5, 30–32]. For example, Cho et al. found that not only the percentages but also the absolute numbers of iNKT cells were obviously lower than that of normal by detection of the number of iNKT cells in 128 SLE patients [5].

Although the decrease of the number of iNKT cells in SLE has been found, whether this decrease is a consequence of the disease or a primary trigger of the disease remains unclear. It has been shown that not only the numbers of iNKT cells were decreased but also the deficiency of iNKT cells correlated closely with the disease activity [5]. The reduced number of iNKT cells can be restored to the normal level when the patient is treated with corticosteroids or rituximab [31, 32]. Consistent with this finding, the correlation between the decrease of iNKT cell numbers and the progression of the disease was observed in lupus murine models [33, 34].

The reduction in iNKT cell numbers could be caused by a defect in their maintenance. The undesirable response of iNKT cells to *α*-galactosylceramide (*α*-GalCer), which is an effective stimulating agent for iNKT cells, has been demonstrated in SLE patients and animal models [30, 35]. When the peripheral blood mononuclear cells (PBMCs) from SLE patients were collected and cocultured with *α*-GalCer, the proliferation of iNKT cells was obviously lower in SLE patients than in the normals. Another study showed that the iNKT cells did not grow when the iNKT cells from SLE were cocultured with *α*-GalCer and normal monocytes, but iNKT cells from the healthy controls expanded successfully under the same conditions [5]. Consistent with this finding, Cho et al. observed that the apoptosis of iNKT cells from SLE patients was increased after incubation with *α*-GalCer for days, but iNKT cells from normal controls did not change [5]. This effect may also result from the observed reduction of iNKT cell numbers in SLE patients.

The response defect of iNKT cells to stimulus may be exacerbated by the compromised expression of costimulatory molecules. CD26 is expressed on the surface of T and B lymphocytes as well as iNKT cells as a costimulatory molecule and is required for connection with other cells. Wong and his colleagues found that CD26 expression was significantly reduced on NKT cells and CD4+ T cells but the expression was not obviously decreased on monocytes, B cells, and CD8+ lymphocytes in SLE patients [36]. Consistent with this finding, the percentage of both CD26+ NKT cells and CD26+CD4+ cells significantly decreased. Because CD26 can link to other cells and play role in activation as a costimulatory molecule, the reduction of NKT cells could result from a defect of costimulatory pathways. The reduced expression of CD26 could consequently hinder NKT cells activation and the proliferation of NKT cells in SLE. Additionally, NKT cells can potentially produce numerous cytokines after being activated by *α*-GalCer. These cells can successfully proliferate by autocrine cytokines [37]. Impaired activation could also influence the cytokines secreted by NKT cells and in turn contribute to the decrease of NKT cells in SLE. The abnormality in the number and the activation of NKT cells in SLE patients in the study indicated that NKT cells may be implicated in the progression of SLE.

## 3. Regulation of Th1 and Th2 Cells by iNKT Cells in SLE

SLE is triggered by autoantibodies aiming at dsDNA and other antigens that ultimately lead to the development of multiorgan failure [38, 39]. The imbalance between Th1 and Th2 cytokines participates in the immunoregulation of SLE [29, 40]. The reduced number of iNKT cells is correlated with the imbalance of Th1/Th2 in SLE patients. In MRL-Fas^lpr/lpr^ mouse model, CD1d gene deletion reduced Th2 cytokine production and failed to affect the production of Th1 cytokines. Tupin and other scholars also found that *α*-GalCer induced Th2 polarization in the aortas of apoE−/− mice [41]. Accordingly, animal models studies have revealed that iNKT cells promote Th2-type cytokine production and allergen-induced airway inflammation through aCD1d-dependent mechanism [42, 43]. Therefore, iNKT cells can regulate the balance of the immune response between Th1 and Th2 cells by secreting cytokines. iNKT cells secrete Th2-biased cytokine production profiles under normal conditions, and, under such conditions, the number of Th2 cells is dominant. Although iNKT cells are considered to maintain the balance of Th1/Th2 cells, the associated regulatory mechanism is not certain. There are a few possible ways to explain how iNKT cells might disturb the differentiation of Th1 and Th2 cells: (1) IL-4 and IL-13 secreted from iNKT cells might directly induce the differentiation of Th2 cells from Th cells; (2) although IL-10/TGF-*β* secreted from iNKT cells might inhibit both Th1 cells and Th2 cells activation, the deviation to Th1 cells or Th2 cells is different under different pathological circumstances; (3) IFN-*γ* might only cause anergy or apoptosis of Th1 cells, leading to the imbalance of Th1/Th2 cells; (4) IL-4, IL-10, and GM-CSF secreted from iNKT cells boost the polarization towards Th2 cells. The inclination may bring about the imbalance of Th1/Th2 cells [44]; (5) some chemokines secreted from iNKT cells promote the generation of DCs; the increased CDs might break the balance of Th1/Th2 cells [44].

## 4. Effect of NKT Cells on IL-17 in SLE

Like other autoimmune diseases, a complex of cytokines is involved in SLE development or pathology. NKT cells can participate in the process in the SLE by secreting cytokines such as IL-17 and IL-21 besides the IL-2, IL-4, IL-6, IL-10, and IFN-*γ* [45, 46]. This finding highlighted the complex of roles that NKT cells play by secreting IL-17, IL-2, and TGF-*β* in SLE [47]. Some studies showed that the level of IL-17 is significantly higher in SLE than the healthy controls [48, 49]. The data suggested IL-17 may play an important role in SLE [50]. It is well known that IL-17 is secreted by CD4 T cells, namely, Th17 cells. However, the current studies showed that iNKT cell can secrete IL-17 and other procytokines in inflammation disease including SLE and RA [51, 52]. Additionally another subtype of NKT cells which can also secrete IL-17 has been identified as IL-17-producing iNKT cells, which have been attracting the attention of more and more scholars since IL-17 plays an important role in infection and tumor as well as inflammatory disease such as SLE [53]. These results indicated that NKT cells are implicated in the pathogenesis of SLE. Even IL-17 production NKT cells are endowed with ability to produce the IL-17; the results of many studies showed the capacity of IL-17-producing iNKT cells is dependent on a proinflammatory environment [19, 54]. These results clarified that NKT cells were complex and pleiotropic. iNKT cells may produce more IL-17 in the proinflammatory environment such as SLE.

## 5. Suppression of Autoantibody Production

SLE is mediated by autoantibodies and controlled by other regulatory lymphocytes cells, including NKT cells. The level of antibodies is related to the disease activity [7]. It has been confirmed that the number of NKT cells and the production were decreased in SLE by many studies. [5, 31, 32], and the number of NKT cells can be recovered after treatment with prednisone [31]. These clinical experiments suggest that NKT cells may ameliorate the state of illness by regulating the level of IgG in SLE [55]. Green et al. identified 65 SLE patients and 45 first-degree relatives of patients with SLE. The result showed that the number of NKT cells was inversely correlated to the level of IgG. The result suggested that NKT cells might control the production of IgG in SLE. On the other hand, the antibodies might also suppress the generation of NKT cells [55].

Consistent with these findings, a number of studies in mouse models also showed that NKT cells possess the regulatory function in SLE. In animal models, Wermeling et al. found a similar phenotype to SLE patients. The manifestations of these lupus-like models included the small number of NKT cells and the high level of IgG [56]. The production of autoantibodies was reduced dramatically when B lymphocytes were cultured with iNKT cells or NK1.1+CD3+ cells were transferred from an MRL-lpr model in vitro [57]. Sireci et al. observed the aged J*α*18-deficient BALB/c mice spontaneously displayed the manifestations of lupus-like models, including the proliferation of the splenic marginal zone B cells [58]. The decrease of CD1d expression on the surface of B cells might cause the production of antibodies and suggested that a direct interaction exists between iNKT cells and B cells. The iNKT cells might transport negative information via CD1d to limit B cell autoreactivity. When B cells come into contact with iNKT cells via CD1d, their capacity to proliferate and produce antibodies decreases. iNKT cells can regulate CD1d+ B cell autoreactivity and prompt production of circulating apoptotic cells; if this role is impaired, an SLE-like phenotype appears [56].

Most studies indicated that NKT cells can suppress the production of IgG, even though some experiments have demonstrated converse conclusions about the relationship between NKT cells and IgG production in humans and mouse models. These contradictory-like results showed that NKT cells have complex functions in the modulation of autoantibody production. More researches will be required to further confirm the influence of NKT cells on the production of autoantibodies.

## 6. Regulation to Dendritic Cells in SLE

Dendritic cells (DCs) mainly consist of conventional dendritic cells (cDCs) and plasmacytoid dendritic cells (pDCs) in humans. DCs are professional antigen presentation cells (APC) which process the antigen and then present to T cells. The activated T cells trigger immune response against self-antigens or exotic pathogens. DCs can capture autoantigens to T cells and make T cell anergy to the autoantigens in certain environment [59]. It is postulated that DCs play an important role in the pathogenesis of SLE due to its double capacity of immune response and immune tolerance [60]. Many relative studies have been performed and the evidence confirmed that DCs contribute to the pathogenesis of SLE. Crispín et al. found the capacity of activation to stimuli is damaged by comparing the DCs from SLE patients and controls. They thought that there were some defects in the process of development and maturation in the DCs of SLE [61]. The consequent studies found the dysfunction of DCs was associated with IRF4-dependent pathways in mouse model [62]. Sisirak et al. found the expression of gene dosage of the pDC-specific transcription factor E2-2 (Tcf4) was abnormal. The results directly proved the dysfunction of DCs in SLE [63]. Fujii et al. found the number of cDCs in the patients with SLE was obviously decreased compared to the normals and there was correlation with disease activity in SLE patients. All the findings pointed out the function changes of DCs in SLE. The changed microenvironment and gene make DCs lose the ability of immune tolerance, which may trigger the occurrence of autoimmune response. The intrinsic defects might result in overactivation of T, B lymphocytes. It has been considered that the iNKT cells play an important role as immune regulator cells. Recently some evidences confirm that iNKT cells can influence the DCs. iNKT cells can reinforce the immune function of DCs by promoting the maturation of DCs [64] and accelerate the ability of DCs to induce the differentiation of T cells by coculturing with DCs in the presence of antigens [65]. The studies illustrated that iNKT cells could affect the function of CDs in certain circulation and strengthened the roles in the appearance of antigens. However, Pilones et al. reported that iNKT cell can defer the action of antitumor T cells by controlling the population of DCs in tumors and draining lymph nodes [66]. iNKT cells can enhance the protective immune response by modulating the function of DCs during* Chlamydia pneumoniae* infection [67]. All the data suggested that iNKT cells have different function in different circumstances. iNKT cell may enhance the immune function of CDs in inflammation or infection and reduce the immune response function of CDs in cancer. We think iNKT cells may arouse the capacity of stimulation of the CDs in SLE. The exogenous antigens trigger the iNKT cells and aggravate the diseases in the environment of infection.

## 7. Target of Treatment

SLE is characterized by mortality rate. The 5-year and 10-year survival rates have been significantly improved as per our knowledge about the pathology of SLE. Although corticosteroids and immunosuppressive agents, such as cyclophosphamide (CTX), are currently the principal treatments, the clinical application of these drugs is limited by their significant side effects [68, 69]. It is necessary to find a therapeutic intervention that specifically targets important immune effector pathways.

NKT cells have emerged as a unique lymphocyte subset that is implicated in many aspects of SLE, such as regulating the balance of different T helper cell populations and suppressing autoantibody production, as described above. Current knowledge supports the hypothesis that NKT cells might play a protective role during the progression of SLE and may become a target for treatment in patients with SLE. However, the functions of NKT cells in animal models might be ambivalent. Some studies showed that anti-CD1d treatment ameliorated manifestations in (NZB×NZW)F1 mice [70], but MRL/lpr mice treated with *α*-GalCer became exacerbation and shortened survival [71]. The healthy models displayed lupus-like manifestations after healthy mice were transferred into iNKT cells from diseased (NZB×NZW)F1 mice [72]. The different results obtained in these studies revealed the complex functions of NKT cells in the pathology of SLE. Despite these concerns, a series of preliminary animal model experiments that aimed to investigate NKT cells in SLE have already been performed. In these studies, *α*-GalCer was identified as a reagent that targets CD1 on NKT cells and is recognized by iNKT cells from a variety of mammalian species, including mouse and humans [73, 74]. *α*-GalCer is considered to be the “gold-standard” for stimulatory ligands [75, 76]. A lupus model, MRL-lpr/lpr mice, exhibits a deficit in iNKT cell numbers and dysfunction. After treatment with *α*-GalCer, the number deficiency was corrected, and inflammatory dermatitis was improved [77]. Zeng et al. [71] reported that when NZB/WF1 mice were treated with anti-CD1d monoclonal antibodies, the titers of some kind of antibodies were decreased and lupus-like expression was ameliorated, but serum levels of IgE were increased. Yang and his colleagues [78] treated BWF1 mice with *α*-GalCer or the vehicle control. The result showed that BWF1 mice exhibited a long-term improvement in renal expression and production of anti-DNA antibodies after treating with *α*-GalCer injections for a few times. These data pieces showed that brief iNKT activation can confer a prolonged protective effect in lupus models. If the lupus-like models were repeatedly treated with *α*-GalCer, the effect on lupus nephritis was poor.

Even though some recent studies show promising results concerning the treatment with *α*-GalCer in human cancer patients [79], we found that the administration of *α*-GalCer can prevent disease, have no effect, or accelerate disease based on the described results from lupus models. It is necessary to perform more investigations into effects of *α*-GalCer-mediated iNKT cell in SLE. Although it is globally accepted that iNKT cells might play a protective role in SLE, some researches showed that these cells may also contribute to autoimmune pathogenesis and even aggravate the disease [71, 80]. Although there are many reasons for the different results obtained in lupus models after treatment with *α*-GalCer, the main reason may be the expression of CD1 on the surface of many lymphocytes besides NKT cells. This factor is expressed primarily on cells of hematopoietic origin, including thymocytes, B cells, macrophages, DCs, and other T cells [81]. Much difficulty remains to be resolved concerning the treatment of SLE with *α*-GalCer, particularly because we do not yet understand the precise mechanisms involved. Thus, *α*-GalCer may be an ideal therapeutic target in SLE, but the use of *α*-GalCer to treat SLE patients requires further study.

## 8. Conclusions

As part of the innate immune system, iNKT cells have attracted attention in the context of immunoregulation. Future advances will improve our comprehension of the function of NKT cells in the pathogenesis of SLE. A few evidences revealed that iNKT cells play an important protective role in the pathogenesis of SLE, even though the mode of action of NKT cells in SLE remains unclear. We believe that iNKT cells perform this regulatory function through the following possible pathways. (1) iNKT cells can secrete a various number of cytokines to maintain the balance of Th1/Th2 cells. Il-4 and IL-13 may directly induce the differentiation of Th2 cells; IL-10 and TGF-*β* might inhibit Th1 cells in certain inflammation condition. (2) Cytokines are directly implicated in the pathogenesis of SLE, such as IL-17-producing iNKT cells. Changes in the number and differences in the subtypes of cytokines are bound to affect the disease. (3) NKT cells linking the innate and adaptive immune responses can suppress B cell proliferation, which results in reduced production of IgG. (4) iNKT cells can regulate the immune response by promoting the maturation of DCs. However, some evidences showed iNKT cells affect the function of DCs depending on the different circulation. Therefore, full understanding of the behavior of NKT cells in SLE will further improve the therapy strategy in future.
